# Validity of the Reaction Time Concealed Information Test in a Prison Sample

**DOI:** 10.3389/fpsyt.2018.00745

**Published:** 2019-01-23

**Authors:** Kristina Suchotzki, Aileen Kakavand, Matthias Gamer

**Affiliations:** Department of Psychology, University of Würzburg, Würzburg, Germany

**Keywords:** concealed information test, deception, lying, reaction times, inmates, forensic sample

## Abstract

Detecting whether a suspect possesses incriminating (e.g., crime-related) information can provide valuable decision aids in court. To this means, the Concealed Information Test (CIT) has been developed and is currently applied on a regular basis in Japan. But whereas research has revealed a high validity of the CIT in student and normal populations, research investigating its validity in forensic samples in scarce. This applies even more to the reaction time-based CIT (RT-CIT), where no such research is available so far. The current study tested the application of the RT-CIT for an imaginary mock crime scenario both in a sample of prisoners (*n* = 27) and a matched control group (*n* = 25). Results revealed a high validity of the RT-CIT for discriminating between crime-related and crime-unrelated information, visible in medium to very high effect sizes for error rates and reaction times. Interestingly, in accordance with theories that criminal offenders may have worse response inhibition capacities and that response inhibition plays a crucial role in the RT-CIT, CIT-effects in the error rates were even elevated in the prisoners compared to the control group. No support for this hypothesis could, however, be found in reaction time CIT-effects. Also, performance in a standard Stroop task, that was conducted to measure executive functioning, did not differ between both groups and no correlation was found between Stroop task performance and performance in the RT-CIT. Despite frequently raised concerns that the RT-CIT may not be applicable in non-student and forensic populations, our results thereby do suggest that such a use may be possible and that effects seem to be quite large. Future research should build up on these findings by increasing the realism of the crime and interrogation situation and by further investigating the replicability and the theoretical substantiation of increased effects in non-student and forensic samples.

## Introduction

Valid lie detection tests would provide valuable means in police interrogations and court, yet unfortunately most lie detection test that have been developed so far are not endorsed by the scientific community. For instance, the Comparison Question Test [also called the Control Question Test, CQT; (1)] has been strongly criticized for its lack of an adequate control condition and its high rate of false positives [i.e., truthful suspects being determined as deceptive; (2, 3)]. Nevertheless, the CQT is the most popular and most commonly applied deception detection test being used by police and secret service in many countries worldwide (e.g., USA and Israel), and in some even admissible as evidence in court (e.g., Belgium). For several years now, scientists have raised their concern about this method and proposed it being replaced with evidence-based tools (4, 5). One of those proposed methods to replace the CQT is the so-called Concealed Information Test [CIT; (6)]. Developed by one of the earliest critics of the CQT, the Concealed Information Test (CIT) does not aim to detect deception, but rather whether a suspect possesses certain incriminating knowledge (therefore, the test had been originally termed the Guilty Knowledge Test). In the CIT, the suspect is presented with a question that only someone with critical crime-knowledge can answer, for instance: “What was the color of the bag that was stolen?” The suspect then receives several possible neutral answers, among which the correct one is hidden, for instance: “Yellow,” “Green,” “Blue,” “Red,” and “Black.” Depending on the CIT version and the dependent measure that is used, the suspect may be instructed to simply listen to those answers or to respond “No” to each of them. The CIT relies on the idea that only a knowledgeable suspect will recognize the correct answer. Note here that therefore the test will never come to the conclusion that a certain suspect is guilty, only that (s)he may be knowledgeable of certain crime aspects. Where this knowledge comes from (e.g., committing the crime, observing the crime, hearsay) needs to be determined in further interrogations. Crucially, it has been found that such recognition leads to measurable changes in different autonomic indices, as for instance an increase in skin conductance, and a decrease in heart rate and respiration for the critical crime knowledge compared to the other neutral answer alternatives (7). No such changes should be observable in an unknowledgeable suspect, for which all alternatives should be equally likely. As the most recent meta-analysis has shown, CIT validity is very promising as evident in a very high effect size (Cohen's *d*) for the differentiation between knowledgeable and unknowledgeable test subjects [*d* = 1.55, *d* = 0.89, and *d* = 1.11 for skin conductance, heart rate and respiration, respectively; (8)].

More recently, it has been shown that behavioral measures such as reaction times (RTs) also show some promise for CIT applications (9, 10). Note that in order to ensure attention to the stimuli, the CIT was for this purpose adapted by asking participants to respond “No” to each of the critical and neutral answer alternatives and to respond “Yes” to a number of designated (crime unrelated) target items (usually via button presses). Using this adapted RT-CIT version also results in a very high effect size, this time calculated as the RT difference between critical and neutral items [*d* = 1.30; for a meta-analysis see (11)]. The main advantage of RT measures in deception detection is their ease of application. For example, they do not require sophisticated equipment (one laptop suffices) or scoring procedures. They do, however, also have a number of potential disadvantages, one of them being that they may not be as easy applicable in populations that differ from the typically studied student and normal populations. Populations such as forensic ones may be less familiar with computerized testing and probably being generally slower may obscure or even eliminate RT CIT-effects. There are also theoretical considerations that may suggest that RT CIT-effects could differ between normal and forensic populations. Whereas the autonomic CIT has been shown to mostly rely on orienting toward familiar or significant stimuli (12–14), there are indications that in the RT-CIT, the requirement to suppress the automatic “Yes” response toward crime related items may also crucially contribute to the effect [i.e., response inhibition; (15–17)]. Importantly, research suggests that response inhibition capacities may be impaired in forensic populations, as well as impulsivity (a trait that has been discussed as being related to response inhibition) increased (18, 19). Thus, instead of being obscured or diminished in forensic populations, the response inhibition account would rather predict the RT CIT-effect to be increased in forensic populations due to an increased difficulty to suppress the unwanted truthful “Yes” response toward critical items. Being the first to employ the RT-CIT in a forensic sample, the current experiment aimed to explore those two contradicting predictions.

## Methods

### Participants

In total, 30 male inmates of a youth detention center in the federal state of Baden-Württemberg in Germany volunteered to take part in the study. The study conformed to the principles expressed in the Declaration of Helsinki. All provided written informed consent. Inclusion criteria for the male control group were, based on the sample of inmates, an age between 16 and 25 years and no education higher than “mittlere Reife” (10 years of formal education, approximately equivalent to the General Certificate of Secondary Education, GCSE). Participants for the control group were recruited through paper and online advertisement (*n* = 6) and via a contact to a vocational school (*n* = 26). All participants from the control group provided written informed consent, and in case they were younger than 18, written informed consent was obtained from the parents. Data of one control participant were exculded because of his higher education. Data of three inmates and six control participants were excluded because they had < 50% trials for one item type in the CIT after exclusion of trials exceeding the response deadline, error trials and RT outliers (see below). The mean age of the remaining 27 inmates was 20.15 years (*SD* = 2.14 years). The mean age of the remaining 25 control participants was 18.88 years (*SD* = 3.17 years). There was no significant age difference between both groups, *t*_(41.74)_ = 1.68, *p* = 0.101, *d* = 0.47.

### Procedure

Testing took place in a quiet room in the youth detention center, in the vocational school building, or at the University. Participants first answered a questionnaire asking for the following demographical data: age, mother tongue, origin, if origin was not German, how long they had already been in Germany, education, type of current employment, and handedness. They then received the instruction that they would see a picture story on the screen of a laptop and they should try to imagine experiencing the depicted scenario. Participants were told to imagine they had to go to the doctor and were in the *waiting room*. They would be alone there and would see a forgotten *handbag*. They would seize the opportunity and look inside the bag. There they would find an *identity card with the name Maria*. They would continue their search and find a *ring* that they would decide to steal. They would still continue and find a *smartphone* that they would also take. Then they would quickly leave the waiting room. Words marked in italics refer to the pictures (i.e., photographs) that were depicted on the screen. Pictures were taken from the internet and can be obtained from the authors upon request (sharing them with the data is not possible due to copyright issues). Participants then saw a short summary of their imaginary activity on the screen: “You were in a WAITING ROOM and stole a RING and a SMARTPHONE from the HANDBAG of MARIA.” Note that the words printed in capital letters were the ones that were later used as critical items in the CIT. The experimenter then asked the participants to repeat those crime details to her, to ensure correct memory of those. Although such an explicit encoding procedure might differ from typical field situations where crime related information is rather encoded incidentally, we chose to use such a procedure to ensure that potential group differences in CIT detection efficacy were not related to group differences in memory for critical items. Now participants were informed that they were suspects of this theft and that they should therefore undergo a lie detection test. For this lie detection test, they further had to memorize five additional words (i.e., the target items). Those words were presented on paper and participants were asked afterwards to write them down to also ensure memory for those. If those were not written down correctly, the words were presented again and this was repeated until all words were remembered correctly. Participants were then instructed to do their best to hide their knowledge of the crime during the following lie detection test. Participants received the instructions for the CIT on the laptop screen. Those instructions specified that they would see words on the screen, one after the other. For each word they should judge as fast as possible, whether they recognized it or not. Importantly, they should only respond “Yes” to the words from the paper list and “No” to all other words. They should further try to always respond as fast and correctly as possible. Responses had to be given via the keyboard (see details below). Participants then performed the CIT. After the CIT, participants were asked to repeat the details from the picture story to the experimenter. They were then asked how motivated they were during the lie detection test (from 1 to 10), how difficult they experienced the test (from 1 to 10) and whether they used any specific strategies to pass the test. They were also asked whether they took any medication or suffered from a physical or mental illness. The experimenter additionally noted a subjective estimation of their German language proficiency (from 1 to 6, 1 being the best according to the German grading system). After this, participants received the instructions for the Stroop task, again on the laptop screen. Those instructions specified that participants would be presented with words in different colors. Their task was to indicate the color of each word while ignoring its meaning. As an example, it was explained that if the word RED would be presented in GREEN color, participants should say “GREEN.” Participants were also told to respond as fast and correctly as possible, as their reaction time would be measured. They were also told that incorrect or too slow responses would result in a black “X” being presented on the screen. Participants then performed the Stroop task. After the Stroop task, participants received another Questionnaire in which they were asked how motivated they were during the Stroop test (from 1 to 10), how difficult they experienced the test (from 1 to 10) and in case they belonged to the control group, whether they were ever found guilty of a crime and if so, what this crime was. Finally, as a measure of trait impulsivity, participants were asked to fill in the Barratt Impulsiveness Scale [BIS-11; (20)]. The BIS-11 comprises 30 items and results in overall values between 30 and 120 with higher values indicating higher trait impulsivity.

### Concealed Information Test

The Concealed Information Test (CIT) was programmed and presented with Inquisit 4. In the CIT, the Question “DO YOU RECOGNIZE THIS WORD” was always presented central in the upper part of the screen. Reminder labels for the two possible responses, “YES” and “NO” were always presented on the left and right lower part of the screen. The position of those labels and thereby the assignment to the “a” and “l” keys on a standard QUERTZ keyboard was counterbalanced between participants. In total, 30 different CIT items were presented centrally on the screen (5 target items, 5 critical items, and 20 neutral items). Note that words instead of pictures were used. A list of all used items can be found on https://osf.io/c5us4/. Each item was presented six times, resulting in 180 trials in total (plus 2 neutral buffer items at the beginning of each test block that were not analyzed). Items were presented in completely randomized order, yet in two blocks each containing each item three times. Between both blocks, participants could take a self-paced break. Each item was presented until a response was given and the inter-trial varied between 500 and 1,000 ms. If participants did not respond after 4,000 ms, the item also disappeared and the words “Too slow!” were presented in red centrally on the screen. No error feedback was given.

### Stroop Task

The Stroop task was presented with Inquisit 4 and the script was taken from the Millisecond test library (http://www.millisecond.com/download/library/). The English instructions and stimuli were translated from English to German und adapted in the experiment script. Responses were given verbally and recorded with the speech recognition function of Inquisit 4. In the Stroop task, the words “red,” “green,” “blue,” and “yellow” were always presented centrally on the screen in one of the four colors. Each color was presented 20 times, 10 times congruent with the corresponding word and 10 times incongruent with one of the other three words (which were chosen randomly). Colors were presented in completely randomized order. They were presented until a response was given and the inter-trial was 200 ms. If participants did not respond after 2,500 ms, the word also disappeared and the next trial started. In case of incorrect responses, error feedback was given in the form of a black “X” presented for 400 ms centrally on the screen.

## Results

Data were analyzed with R and raw data as well as analysis scripts can be accessed on https://osf.io/c5us4/. To compare demographics between both groups, Fisher's Exact Test for Count Data was used, testing the null hypothesis that the odds ratio is equal to one. Analysis steps for the CIT were as follows. First, trials exceeding the response deadline were excluded (2.78%). Mean error rates were computed separately for probes and irrelevant items and analyzed with a two (Group: inmates vs. control) × 2 (Item: critical vs. neutral) mixed ANOVA. Before conducting the same 2 × 2 ANOVA on RTs, error trials (9.40%) and RT outliers (2.40%; RTs >2.5 *SD*s from the mean per subject and item type) were removed. For the analysis of the Stroop task the preprogrammed standard script as implemented in the experimental task taken from http://www.millisecond.com/download/library/ was used. Here, error trials (3.39%) were removed, before mean RTs were computed separately for congruent and incongruent trials and analyzed with a two (Group: inmates vs. control) × 2 (Congruency: congruent vs. incongruent) repeated measures ANOVA.

For ANOVA effects, η_*p*_^2^ was calculated as a measure of effect size. For follow-up *t*-tests, the standardized mean difference *d* was calculated, with 0.20, 0.50, and 0.80 as thresholds for “small,” “moderate,” and “large” effects (21). When *d* was computed for dependent samples, it was corrected for inter-correlations (22, 23).

### Demographics and Questionnaire

An overview of the demographic data is given in Table 1.

**Table 1:** Demographic data of inmates and control group.

	**Inmates**	**Control**	***p***
**ORIGIN**			1
Germany	23	21	
Other	4	4	
**MIGRATION BACKGROUND**			1
Yes	16	15	
No	11	10	
**MOTHER TONGUE**			1
German	17	15	
Other	10	10	
**HIGHEST EDUCATION**			< 0.001^***^
No diploma	2	9	
“Hauptschule” (9 years of formal education)	20	6	
“Realschule” (10 years of formal education)	5	10	
**APPRENTICESHIP**			0.046^*^
Yes	0	4	
No	27	21	
**CURRENT EMPLOYMENT**			0.344
School	7	9	
Apprentice	19	13	
Employed	1	3	
**HANDEDNESS**			1
Left	3	2	
Right	24	23	

*p-values reported two-tailed*.

* *p < 0.05*,

*** *p < 0.001*.

Ratings of the estimated German language proficiency (from 1 to 6, 1 being the best according to the German grading system, rated by the experimenter) as well as the number of remembered crime-related items and the motivation and perceived difficulty of the CIT and the Stroop task can be found in Table 2.

**Table 2:** Questionnaire data of inmates and control group.

	**Inmates**	**Control**	***t***	***df***	***p***	***d***
Language proficiency	1.30 (0.47)	1.04 (0.20)	2.61	35.86	0.013*	0.72
Number of remembered Items	4.70 (0.72)	4.68 (0.63)	0.13	49.79	0.900	0.04
Motivation CIT	8.44 (1.70)	8.24 (2.05)	0.39	46.75	0.698	0.11
Difficulty CIT	4.67 (2.39)	4.60 (2.10)	0.11	49.88	0.916	0.03
Motivation Stroop	8.44 (1.72)	8.64 (1.71)	0.41	49.75	0.682	0.11
Difficulty Stroop	4.63 (2.68)	4.72 (2.30)	0.13	49.74	0.896	0.04

Standard deviations are given in brackets. p-values reported two-tailed. ^*^p < 0.05.

### Results CIT

The mean error rate for all four conditions can be found in Table 3. The 2 × 2 ANOVA on the error rate revealed a significant main effect of Group, *F*_(1, 50)_ = 6.06, *p* = 0.017, *n*_*p*_^2^ = 0.11, with a higher error rate for the inmates compared to the control group. It also revealed a significant main effect of Item, *F*_(1, 50)_ = 24.43, *p* < 0.001, *n*_*p*_^2^ = 0.33, with a higher error rate for critical compared to neutral items. These effects were qualified by a significant interaction of Group x Item, *F*_(1, 50)_ = 5.90, *p* = 0.019, *n*_*p*_^2^ = 0.11, with a larger CIT-effect (i.e., differences between critical and neutral items) in the inmates *t*_(26)_ = 4.30, *p* < 0.001, *d* = 0.83, compared to the control group, *t*_(24)_ = 2.66, *p* = 0.014, *d* = 0.53.

**Table 3:** Mean error rates and RTs in all four experimental CIT conditions.

	**Error rate (in %)**		**Reaction times (in ms)**	
	**Inmates**	**Control**	**Inmates**	**Control**
Critical items	11.64 (13.30)	4.41 (6.35)	1033.02 (204.55)	1016.76 (239.43)
Neutral items	1.00 (1.62)	0.78 (1.35)	744.49 (118.41)	785.91 (172.40)
*d*_CIT−effect_	0.83	0.53	1.86	1.48

*Standard deviations are given in brackets. d_CIT−effect_ values refer to the difference between critical and neutral items within each group*.

The mean RTs for all four conditions can be found in Table 3. The 2 × 2 ANOVA on the RTs revealed only a significant main effect of Item, *F*_(1, 50)_ = 144.24, *p* < 0.001, *n*_*p*_^2^ = 0.74, with longer RTs for critical compared to neutral items. Neither the main effect of Group, *F*_(1, 50)_ = 0.07, *p* = 0.792, *n*_*p*_^2^ < 0.01, nor the interaction of Group x Item, *F*_(1, 50)_ = 1.78, *p* = 0.188, *n*_*p*_^2^ = 0.03, were statistically significant.

### Results BIS-11 and Stroop Task

The mean BIS-11 value in the inmates group was *M* = 65.89 (*SD* = 8.42) and *M* = 65.92 (*SD* = 8.24) in the control group, with no significant difference between both groups, *t*_(49.84)_ = 0.01, *p* = 0.989, *d* = 0.00. Cronbach's α for the BIS-11 was not very high with 0.66. There were also no significant differences between both groups in any of the BIS-11 subscales, all *p*'s > 0.05. The mean RTs (in ms) for all four conditions in the Stroop Task were *M* = 735.27 (*SD* = 97.57) in the inmates group in the congruent trials, *M* = 919.11 (*SD* = 128.52) in the inmates group in the incongruent trials, *M* = 807.95 (*SD* = 112.44) in the control group in the congruent trials, and *M* = 968.20 (*SD* = 138.84) in the control group in the incongruent trials. The 2 × 2 ANOVA revealed only a significant main effect of Congruency, *F*_(1, 50)_ = 252.35, *p* < 0.001, *n*${}_{\text{p}}^{2}$ = 0.83, with longer RTs for incongruent compared to congruent trials. Neither the main effect of Group, *F*_(1, 50)_ = 3.73, *p* = 0.059, *n*_*p*_^2^ = 0.07, nor the interaction of Group × Congruency, *F*_(1, 50)_ = 1.19, *p* = 0.281, *n*_*p*_^2^ = 0.02, were statistically significant.

### Correlations

Correlations between both CIT-effects, Stroop effects and participants' scores in the BIS-11 are shown in Table 4. As can be seen, there was only a significant correlation between the CIT-effects in the error rate and the RTs, but no significant correlations between those and the Stroop effects or the BIS-11 values. Note that based on the suggestion of a reviewer, we also checked the intercorrelations between CIT-effects and Stroop effects and the BIS-11 subscales (while controlling for multiple testing due to the exploratory nature of those analyses), which also revealed no significant correlations.

**Table 4:** Correlations (*r*) between CIT-effects, Stroop-effects and BIS-11.

**Measure**	**ER CIT- effect**	**RT CIT-effect**	**Stroop effect**	**BIS-11**
ER CIT-effect	–	–	–	–
RT CIT-effect	0.51^***^	–	–	–
Stroop effect	0.04	−0.14	–	–
BIS-11	−0.17	−0.16	0.15	–

*p-values reported two-tailed*.

*** *= p < 0.001*.

## Discussion

The aim of the current study was to explore the applicability of the RT-CIT in a sample different from the samples usually investigated in experimental research. This is particularly important as the latter differ fundamentally from the ones in which a CIT would ultimately be applied on and even currently is in field investigations in Japan. Nevertheless, studies examining the CIT in forensic samples are very scarce and particularly for the RT-CIT even non-existing. In the current study, we therefore recruited inmates of a youth detention center to complete an imaginary mock crime and afterwards an RT-CIT. As a control group, we recruited a sample that we tried to match as closely as possible regarding age and education background. Note that thereby also the control group differs from the student samples usually investigated in psychological research.

The first notable result is that in both samples, the RT-CIT produced medium to large effects in error rate and RTs. Effects were larger in the RTs than in the error rate, which is in accordance with results usually obtained with the RT-CIT [e.g., (10, 24–26)]. This result is of course very promising for applied contexts and speaks against the argument that the RT-CIT may not be applicable in samples that are less familiar with computerized tests. Note here that one adaptation that we made is that instead of the typically used response deadlines of 800 or 1,000 ms (9, 10, 25, 26), we used a longer response deadline of 2,500 ms. This was primarily done to ensure that the RT-CIT would also be applicable in participants with generally slower responding. The use of short response deadlines does therefore not seem mandatory to obtain stable RT-CIT effects and the mean RTs in our samples indicate that a shorter response deadline may still have been applicable. Such a shorter response deadline would also be desirable as it makes it harder for suspects to strategically slow down responses and employ so-called countermeasures (see also below).

The second notable result is that at least in the error rates, CIT-effects were even stronger in the inmate group compared to the control group. Although numerically also the case for the RTs, this difference did not become significant. This allows a number of possible explanations. First, the absence of significant group differences in the RTs may simply represent a power issue and may not necessarily indicate a genuine dissociation between both measures. However, even though we cannot ensure an absence of group differences in RTs, our data at least indicate that such group differences seem to be larger for error rates as compared to RTs. Second, the current pattern of results might indicate differences between both groups in their speed accuracy trade-off. Thus, control participants might have concentrated more on avoiding errors even at the expense of longer response latencies than inmates. Whereas, the generally higher error rate for the inmates compared to the control group substantiates this notion, the absence of reversed general effects for RTs speaks against such shift of the response criterion. Of course, we also cannot exclude from our data that the difference between both groups in the error rate may constitute a chance finding, and a replication of our finding, preferably by a different research group, would be highly desirable. Note also that as mentioned above, our control group was deliberately designed to be closely matched to our inmate group, as we wanted to isolate differences related to the forensic background of the inmates and minimize differences related to age or education. One would, however, expect differences to be even larger between forensic samples and the ones typically tested in experimental research, a hypothesis that would be worth pursuing in future research. Such research should also incorporate a formal assessment of IQ, instead of only assessing education levels.

Importantly, our data provides no support for the hypothesis that differences in response inhibition capacities or impulsivity may explain larger CIT-effects in our forensic sample. While based on previous findings it is not so surprising that we did not find any correlation between our behavioral measure of executive functioning (i.e., the Stroop task) and our impulsivity measure [i.e., the BIS-11; (27–29)], it was unexpected that we even failed to observe differences in those measures between both groups. One explanation here may be that despite our matching not having succeeded perfectly (with differences in education and language proficiency), groups were still very similar. Also here, increasing group differences between the forensic and the control group may increase differences in executive functioning and impulsivity traits between both groups. The absence of a correlation between the BIS-11 and the Stroop effect with both CIT-effects, respectively, does, however, question the hypothesis that differences in those constructs may explain any differences in the size of CIT-effects. Note that this is against theoretical accounts and previous results indicating a substantial contribution of failures of response inhibition to deception and the RT CIT-effect (16, 17). It is, however, noteworthy that despite the popularity of this account, results so far are still mixed [see e.g., (30, 31)] and one fundamental challenge that has still received insufficient attention would be to better isolate which of the different facets of executive functioning [working memory vs. response inhibition vs. task switching (15, 32)] or even response inhibition [e.g., interference inhibition vs. action cancelation; measured with e.g., Stroop or Stop-Signal tasks; (33)] is the one that actually contributes to the CIT-effect.

As mentioned above, our findings seem promising for applied contexts, although it should be kept in mind here that so far, the CIT is only rarely applied and accepted in court. An exception is Japan where ~5,000 CIT examinations are carried out by the police each year (34). However, CIT examinations are based on recordings of autonomic nervous system activity in Japan and not on behavioral measures as in the current study. Yet even with the autonomic CIT, experimental research in forensic samples (35, 36) or field investigations in such populations (37–39) are still very rare. Filling this gap seems important for two reasons. First, it would provide information on the validity of the CIT in the population in which it is actually applied, providing the basis for a more informed debate on whether this test should be applied and, as supported by many CIT researchers (4) replace currently used invalid lie detection methods (e.g., the CQT). Second, it would be very interesting from a theoretical perspective, as it has been argued that the autonomic and the RT-CIT differ with regard to their underlying psychological mechanisms [orienting vs. response inhibition (16)]. Following this line of arguments, one would expect the autonomic CIT to be less affected by the specific population than the RT-CIT. Another interesting question to pursue would be to what degree different populations may differ with regard to their potential countermeasure use. Countermeasures are deliberate strategies taken by suspects in order to systematically influence their test outcome and increase their chance of being classified innocent (40). The likelihood and the ability to successfully employ countermeasures may be dependent on many variables (e.g., experience with the CIT and/or computer-based testing, education) and may therefore differ between populations. On a related note, it has also often been hypothesized that people with psychopathic personality traits, whose prevalence is higher in forensic samples, may have better deception skills (41–45), which may result in smaller CIT effects or an increased likelihood to successfully implement countermeasures. Future research should therefore also aim to employ assessments of psychopathy.

One of the limitations of the current study is certainly the use of an imaginary instead of an actual mock crime scenario. The reasons that we employed an imaginary one were to be independent of the specific locations the experiment was run at (e.g., the detection center and the school) and ethical considerations, as we did not want to give the impression of furthering “illegal” behavior in a forensic population, even if it was only a role play (as is usually the case in mock crimes). Future research should, however, aim at increasing the realism of the crime and interrogation situation, in order to obtain information to what degree for instance a larger emotional involvement may impact crime-related memory in forensic populations (46). Such a more ecologically valid crime could for instance involve an actual mock crime, which should of course be very carefully instructed as role play in a prison sample. The same is true for increasing the realism of the interrogation situation, in which the experimenter could be introduced as actual police interrogator, which for instance conducts the test for training purposes.

To sum up, the current study provides a first crucial step toward an investigation of the RT-CIT in a forensic population. It indicates the usability of the RT-CIT in such a population, with even some support that effects may even be stronger. Further research should continue this challenge by investigating the replicability of those effects as well was their theoretical substantiation.

## Ethics Statement

The ethics committee of the Department of Psychology of Wuerzburg University usually does not require ethical approval for single studies using well-established (also slightly adapted) experimental protocols and procedures that have obtained ethical approval before (as is the case in our study). The study was discussed and approved by the responsible at the JVA Adelsheim, in which we recruited part of our sample.

## Author Contributions

KS was involved in the study conception and the design, the analysis and interpretation of the data, and the writing of the manuscript. AK was involved in the recruitment of the participants, the collection and interpretation of the data, and the critical revision of the manuscript. MG was involved in the study conception and the design, the interpretation of the data, and the critical revision of the manuscript.

### Conflict of Interest Statement

The authors declare that the research was conducted in the absence of any commercial or financial relationships that could be construed as a potential conflict of interest.
